# Short-term effects of air pollution exposure on the risk of preterm birth in Xi’an, China

**DOI:** 10.1080/07853890.2022.2163282

**Published:** 2023-01-04

**Authors:** Liren Yang, Guilan Xie, Wenfang Yang, Ruiqi Wang, Boxing Zhang, Mengmeng Xu, Landi Sun, Xu Xu, Wanwan Xiang, Xiaoyi Cui, Yiwen Luo, Mei Chun Chung

**Affiliations:** a Department of Obstetrics and Gynecology, Maternal & Child Health Center, The First Affiliated Hospital of Xi’an Jiaotong University, Xi’an, P.R. China; b School of Public Health, Xi’an Jiaotong University Health Science Center, Xi’an, P.R. China; c The National Medical Center Office, The First Affiliated Hospital of Xi’an Jiaotong University, Xi’an, P.R. China; d College of Public Health, Zhengzhou University, Zhengzhou, P.R. China; e College of Nursing, Peking University Health Science Center, Beijing, P.R. China; f Division of Nutrition Epidemiology and Data Science, Friedman School of Nutrition Science and Policy, Tufts University, Boston, MA, USA

**Keywords:** Air pollution, preterm birth, short-term, time series

## Abstract

**Introduction:**

Long-term exposure to air pollution is known to be harmful to preterm birth (PTB), but little is known about the short-term effects. This study aims to quantify the short-term effect of particulate matter with aerodynamic diameter ≤ 2.5 μm (PM_2.5_), ≤10 μm (PM_10_) and nitrogen dioxide (NO_2_) on PTB.

**Materials and methods:**

A total of 18,826 singleton PTBs were collected during the study period. Poisson regression model combined with the distributed lag non-linear model was applied to evaluate the short-term effects of PTBs and air pollutants.

**Results:**

Maternal exposure to NO_2_ was significantly associated increased risk of PTB at Lag1 (RR: 1.025, 95%CI: 1.003–1.047). In the moving average model, maternal exposure to NO_2_ significantly increased the risk of PTB at Lag01 (RR: 1.029, 95%CI: 1.004–1.054). In the cumulative model, maternal exposure to NO_2_ significant increased the risk of PTB at Cum01 (RR:1.026, 95%CI: 1.002–1.051), Cum02 (RR: 1.030, 95%CI: 1.003–1.059), and Cum03 (RR: 1.033, 95%CI: 1.002–1.066). The effects of PM_2.5_, PM_10_ and NO_2_ on PTB were significant and greater in the cold season than the warm season.

**Conclusions:**

Maternal exposure to NO_2_, PM_2.5_ and PM_10_ before delivery has a significant risk for PTB, particularly in the cold season.

**Key messages**
- Maternal exposure to NO_2_ was significant associated with an increased risk of preterm birth at the day 1 before delivery.
- Particle matter (PM_2.5_ and PM_10_) showed a significant short-term effect on preterm birth in the cold season.
- The effects of air pollutants on preterm birth was greater in the cold season compared with the warm season.

## Introduction

The rates of preterm birth (PTB, <37 completed weeks’ gestation) [1] have been increasing since 2000, globally, more than one in 10 babies were born preterm [2]. According to the WHO report in 2018, across 184 countries, the rate of PTB ranged from 5% to 18%, and every year, an estimated 15 million babies are born preterm, and this number is rising [3]. The estimated global PTB rate for 2014 was 10.6% (uncertainty interval 9.0–12.0), China accounted for 1.2 million (7.8%) of PTB in 2014 [2]. PTB complications are the main cause of death among children under 5 years of age, responsible for approximately 1 million deaths in 2015 [4]. Similarly, complications related to PTB were the main causes of child under-five mortality and one of the most common causes of child death in China [4,5]. PTB also significantly increased the risk of hospitalization for illness during childhood [6].

PTB occurs for a variety of reasons [7]. A better understanding of the causes and mechanisms could advance the development of solutions to prevent PTB. With the emerging concern in air pollutant and its health effects [8], some studies have indicated exposure to air pollution during late pregnancy maybe cause inflammation, thus changing the blood viscosity [9], then affect placenta hypoperfusion and result in PTB [10]. A lot of researches reported maternal consistent exposure to air pollution increased the risk of PTB [11–13], and the sensitive trimesters for pollutants were found [14–16]. Sun and Malley et al. found that gestational exposure to particulate matter was significantly associated with the occurrence of PTB [13,17]. Similarly, some researchers suggested that there may be a short-term effect of air pollution on PTB. To our knowledge, few studies have explored the short-term effects of air pollution on PTB, employing time-series, but findings remained inconclusive [18–21].

The air pollution level of China in 2017 exceed the WHO Air Quality Guideline, with 81% living in regions exceeding the WHO Interim Target 1, and air pollution remain a crucial risk factor for health [22]. Therefore, in this study, the distributed lag linear model (DLM) and generalized additive model (GAM) were used to assess the association between the short-term exposure to air pollutants and the risk of PTB in Xi’an, China, aim to explore the short-term effects of air pollution on PTB better.

## Materials and methods

### Study area

Xi’an is the largest central city in northwest China covering an area of 10,752 km^2^ with a population of 10.20 million in 2019. Located in the middle of Guanzhong Plain, the city consists of 13 districts/counties, ranging in size from 23.37 km^2^ to 2,945.20 km^2^. The 13 districts and 13 monitoring stations included in this study are shown in Supplemental Figure S1. From 2015 to 2018, among the 1459 days monitored, the number of good days was 811(55.59%), light pollution was 397(27.21%), moderate pollution was 128(8.77%), heavy pollution was 95(6.51%), and severe pollution was 28(1.92%) according to the Chinese National Air Quality Standards of GB3095-2012 (http://xaepb.xa.gov.cn/).

### Study population

A total of 423,526 live singleton births whose mothers resided in Xi’an city were collected from 1 January 2015 to 31 December 2018. Eligible live births with gestational age < complete 37 weeks were defined as PTB. A total of 18,826 (4.45%) preterm single births whose gestational ages within the range of 20–36^+6 ^weeks and mothers age within the range of 15–50 years were included in the analysis. For each birth, we extracted data on the address of residence, maternal age, time of birth, gestational age (GA), birth weight, etc. All the data on the birth certificate has been audited by the delivery personnel, the parents and the issuing personnel. This study was approved by the Medical Ethics Committee of the First Affiliated Hospital of Xi’an Jiaotong University (No. XJTU1AF2020LSK-261) based on the institution’s ethical standards and the ethical standards of the Declaration of Helsinki. Due to the data analysed of this study were collected without any individual identifiers, the need for informed consent was exempted from Medical Ethics Committee.

### Exposure assessment

Real-time data of PM_2.5_, PM_10_ and NO_2_ exposure from 2015 to 2018 were provided by the Chinese Air Quality Online Monitoring and Analysis Platform (https://www.aqistudy.cn/), which covered 13 air pollution monitoring sites in Xi’an city of Shaanxi province. To improve the accuracy of air pollution exposure assessment, maternal exposure was assessed on a county basis. First, we used the addresses of mothers with PTB to determine the median centre of the PTB in each county. Then the daily 24-h average concentration of PM_2.5_, PM_10_ and NO_2_ exposure of each centre was evaluated by inverse distance weighting (IDW) method with powers of 2, and the exposure levels were estimated as the exposure of the population in the county. When the missing value of monitoring station concentration on the day was greater than 4, the pollution value of the day was assigned as the missing value. To evaluate the robustness of the air pollution assessment, we also used the mean concentration of Xi’an and IDW with powers of 1 to assess the air pollution exposure. The daily mean temperature (°C), atmospheric pressure (hPa), and relative humidity (%) data of the whole city were obtained from the Xi’an Meteorological Bureau websites (http://sn.cma.gov.cn/dsqx/xaqx/). We match daily air pollutants and weather records to the birth records by exact date of birth. The missing values of air pollution and meteorological variables were dealt with seasonal adjustment then linear interpolation during the analysis. Referring to previous studies [18–21], our study mainly considered the effect of air pollution seven days before delivery on preterm birth.

### Data analysis

The count of daily PTB follows a quasi-Poisson distribution [23]. The quasi-Poisson regression model combined with the GAM and DLM was applied to simultaneously investigate the dose-response and delayed effects of air pollution on daily PTB [24]. This methodology of DLM was based on a ‘cross-basis’ function, which allowed the linear effect of daily PTB variation at each lag and the non-linear effects across lag days to be estimated [23]. In this study, single-day lag model from the current day (Lag0) to seven days prior (Lag7) was established by GAM, moving average model for the current day and the previous seven days (from Lag01 to Lag07) was established with Gam, and cumulative model from the current day (Cum01) to seven days prior (Cum07) was established with DLM to estimate the cumulative overall lag effects of each day. The analysis was carried out in two stages. In the first stage, the linear effects of air pollution in each county were estimated using the quasi-Poisson regression model combined with GAM and DLM for lags of 0–7 days prior to delivery. In the second stage, we pooled the estimated county-specific relative ratios using a random effects model with DerSimonian-Laird estimator method.

The single-models used for the analysis in the first stage were as follows:

GAM:

$$\mathrm{Log}[E(Y_{ti})]=\mathrm{intercept}+{\beta X}_{ti}+s(\mathrm{Temperature},\mathrm{df}=3)+s(\mathrm{time},\mathrm{df}=6)+s({\mathrm{RH}}_{t},\mathrm{df}=3)+\beta {\mathrm{Dow}}_{t}+\beta {\mathrm{Number}}_{ti}+s({\mathrm{Month}}_{t},\mathrm{df}=5)+\beta P_{t}$$

DLM:

$$\mathrm{Log}[E(Y_{ti})]=\mathrm{intercept}+{cb}_{i}(\text{Air pollutants})+ns(\mathrm{Temperature},\mathrm{df}=3)+ns(\mathrm{time},\mathrm{df}=6)+ns({\mathrm{RH}}_{t},\mathrm{df}=3)+\beta {\mathrm{Dow}}_{t}+\beta {\mathrm{Number}}_{ti}+ns({\mathrm{Month}}_{t},\mathrm{df}=5)+\beta P_{t}$$

The double-models used for the analysis were as follows:

$$\mathrm{Log}[E(Y_{ti})]=\mathrm{intercept}+{\beta X}_{1ti}+{\beta X}_{2ti}+s(\mathrm{Temperature},\mathrm{df}=3)+s(\mathrm{time},\mathrm{df}=6)+s({\mathrm{RH}}_{t},\mathrm{df}=3)+\beta {\mathrm{Dow}}_{t}+\beta {\mathrm{Number}}_{ti}+s({\mathrm{Month}}_{t},\mathrm{df}=5)+\beta P_{t}$$

Where E(*Y_t_*) denotes the expected number of daily PTB on day t of county i, *β* is the regression coefficient of exposure, X_ti_ is the concentration of pollutant on day t of county i, *cb*i means the ‘cross-basis’ function of county i, *ns* represents the natural spline function, time represents the long-term temporal trend and the seasonal trend, Dow*_t_* represents the week on day t, RH*_t_* represents the daily relative humidity on day t, Number represents the daily number of births on day t of county i, Month*_t_* represents the month on day t, P_t_ represents the atmospheric pressure (hectopascal, hPa) on day t. Because the number of births in the population varies by year, we included ‘Number’ in the model to offset the change during the study period [18].

Based on the quasi-Akaike information criterion (QAIC) for quasi-Poisson models [23,25], we evaluated the optimal df for time with 6° and temperature with 3° to control the seasonal and long-term trends and the effect of temperature for each county, and we found that a line function for RH*_t_*, Dow*_t_*, Number*_t_*, and Month*_t_* produced the best model fit. We used a line function to evaluate dose-response relationship and a natural spline function with 3 df for each county for lag seven days with the lowest QAIC value. We examined the risks for an IQR increase of PM_2.5_, PM_10_ and NO_2_ on daily PTB for different lags.

We applied a double-pollutant model to estimate the effect of interaction between each pollutant on the risk of PTB. Subgroup analysis was conducted according to season. The seasons were divided into warm season and cold season according to the temperature of Xi’an [26]. April and May belonged to the spring; June to August belonged to summer; September and October belonged to autumn, and November to March belonged to winter. In this article, the three seasons of spring, summer, and autumn were combined into the warm season. We performed the following sensitivity analyses: (1) analysis of mother’s age restricted to 20–40 years, to assess whether the results of the primary analysis were driven by maternal age; (2) analysis of GA restricted to 24–36^+6 ^weeks and 28–36^+6 ^weeks, to assess whether the results of the primary analysis were driven by medical reasons which cause PTB. All statistical tests were two-sided and values of *p* < .05 were considered statistically significant. The ‘dlnm’ and ‘mgcv’ package in R software version 4.0.5 was utilized to fit all the models [23,27], the ‘metafor’ package was utilized to pool effect values.

## Results

**Figure 1.**
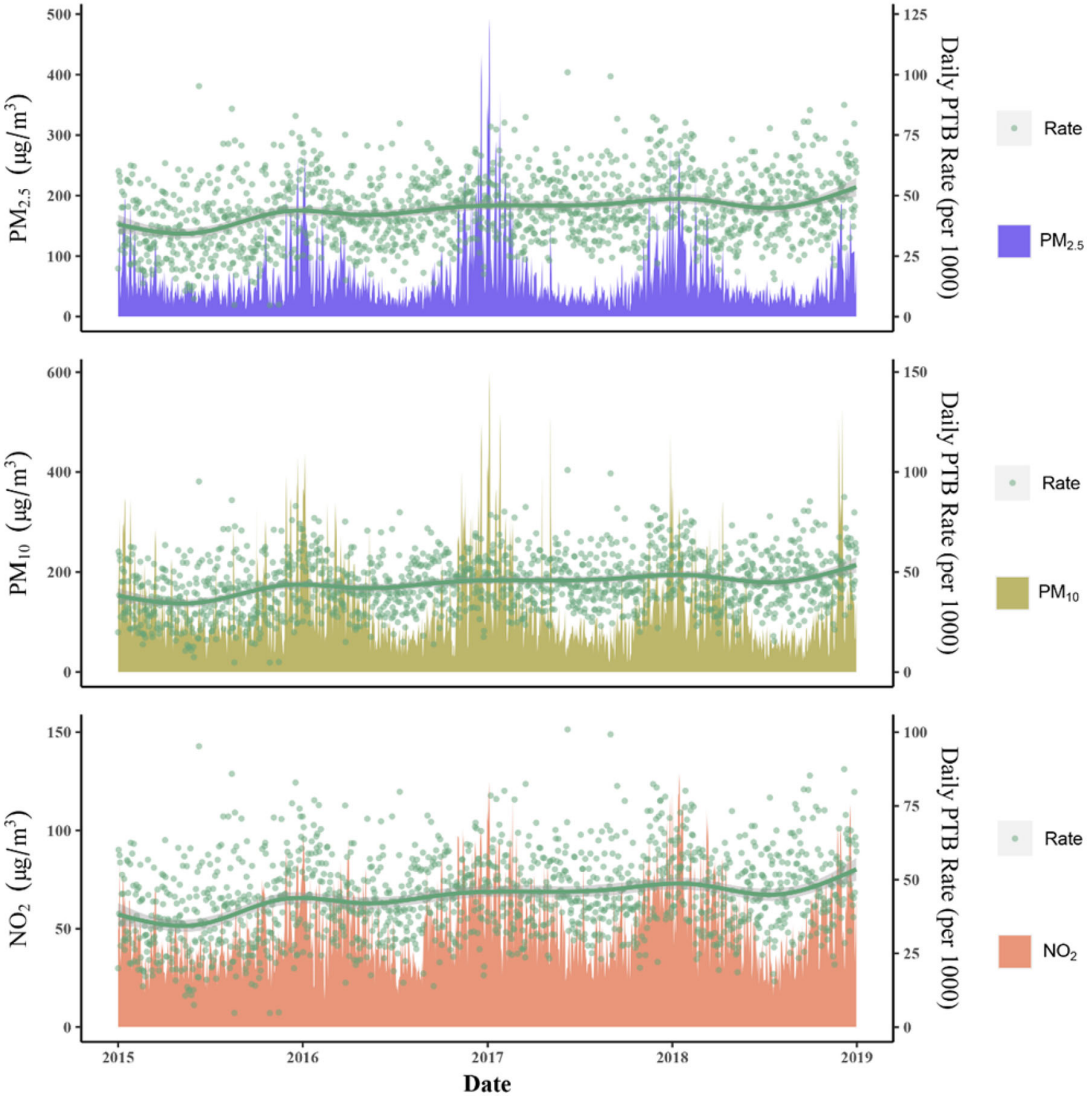
The distribution of air pollutant concentrations and daily PTB rate in Xi’an, China during 2015–2018.

Detailed summary statistics of the study population were shown in Table 1. There were 423,526 single births in Xi’an between 2015 and 2018 including 18,826 (4.45%) single PTB. The mean of daily births and daily PTB of each county ranged from 6.95 to 45.84 and 0.28(4.03%) to 2.29(5.00%), respectively. The distribution of air pollutants in Xi’an was displayed in Figure 1, the distribution of meteorological variables was displayed in Supplemental Figure S2. The average concentrations of PM_2.5_, PM_10_ and NO_2_ of 13 counties were ranged from 60.68 to 69.65 μg/m^3^, from 109.60 to 130.26 μg/m^3^, and from 41.53 to 56.29 μg/m^3^, respectively. A total of 1455 observation days were available for PM_2.5_, PM_10_ and NO_2_ with 5 missing data, and there were 5 missing data of temperature and relative humidity, respectively. The mean temperature, relative humidity and pressure were 16.30 °C (ranging from −6.70 to 35.70 °C), 59.00% (ranging from 17.50 to 98.00%), and 968.90 hPa (ranging from 948.70 to 1000.60), respectively.

**Table 1.** Summary statistics of study population.

County	Number of births		PTB				GDP (100 million yuan)
	Total (423,526)	Daily mean	Total (18,826)	Mean	Min	Max	
Baqiao	34,604	23.69	1,627	1.11	0	7	387.95
Beilin	26,950	18.45	2,010	1.38	0	11	814.54
Changan	55,439	37.95	2,303	1.58	1	8	705.51
Huyi	23,933	16.38	819	0.56	0	5	183.22
Gaoling	17,200	11.77	657	0.45	0	5	338.25
Lantian	18,653	12.77	782	0.53	0	5	132.43
Lianhu	32,090	21.96	1,472	1.01	0	5	691.43
Lintong	26,627	18.23	820	0.56	0	5	208.69
Weiyang	63,273	43.31	2,827	1.94	1	9	825.62
Xincheng	19,412	13.89	950	0.65	0	6	575.35
Yanliang	10,150	6.95	414	0.28	0	3	218.24
Yanta	66,965	45.84	3,346	2.29	1	11	1419.92
Zhouzhi	28,230	19.32	799	0.55	0	4	124.96

*Note:* Min: minimum; Max: maximum.

**Figure 2.**
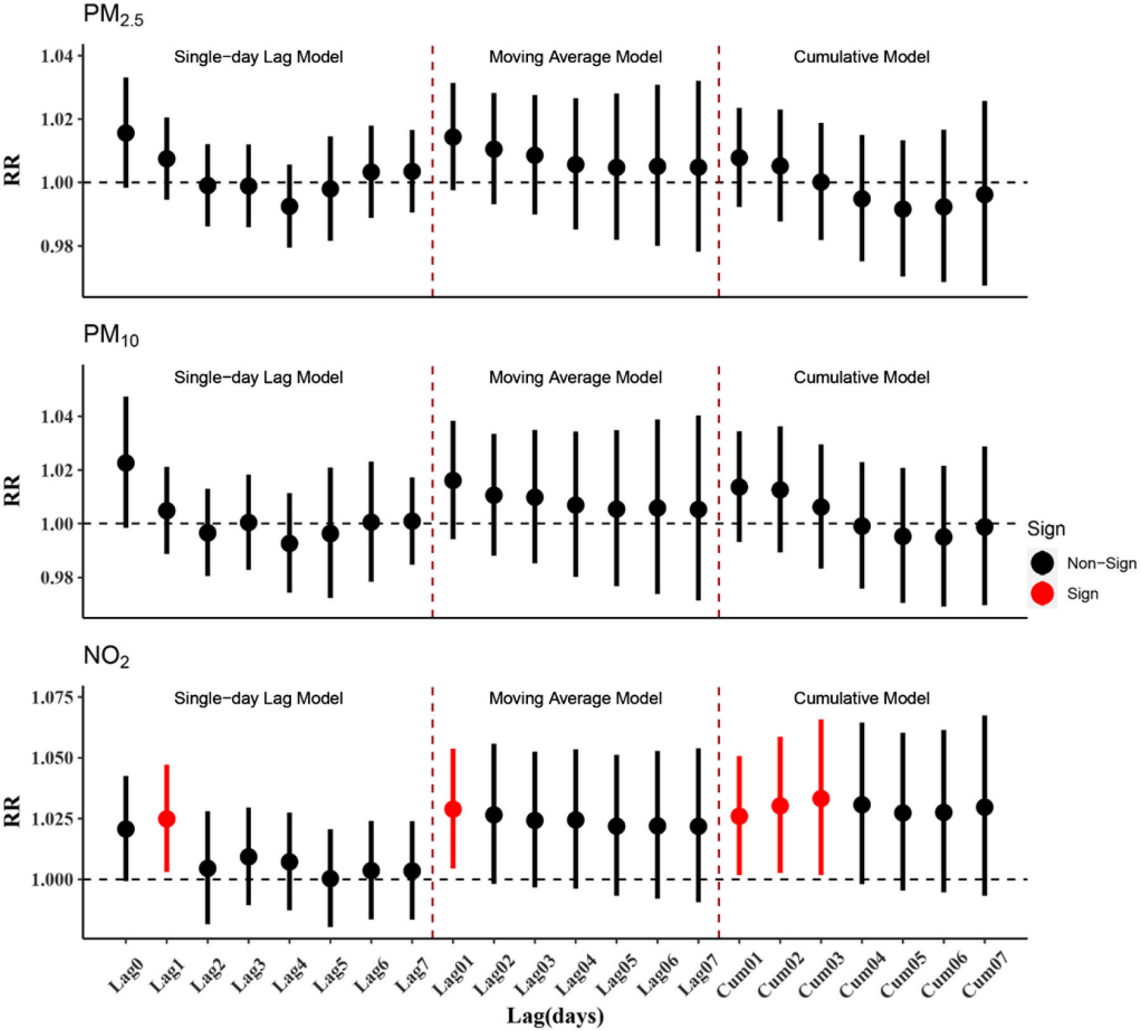
Estimated relative risks (RRs) and 95% confidence intervals (CIs) of PTB for an IQR μg/m^3^ increase of PM_2.5_, PM_10_ and NO_2_.

Figure 2 shows that in the single-day lag model, maternal exposure to NO_2_ was significant associated with an increased risk of PTB at Lag1 (RR: 1.025, 95%CI: 1.003–1.047). In the moving average model, maternal exposure to NO_2_ was significant associated with an increased risk of PTB at Lag01 (RR: 1.029, 95%CI: 1.004–1.054). In the cumulative model, maternal exposure to NO_2_ was significant associated with an increased risk of PTB at Cum01 (RR: 1.026, 95%CI: 1.002–1.051), Cum02 (RR: 1.030, 95%CI: 1.003–1.059), and Cum03 (RR: 1.033, 95%CI: 1.002–1.066). The I^2^ of pooled estimates ranged from 0 to 49.567 for single-day lag model, moving average model and cumulative model (Supplemental Table S1).

**Figure 3.**
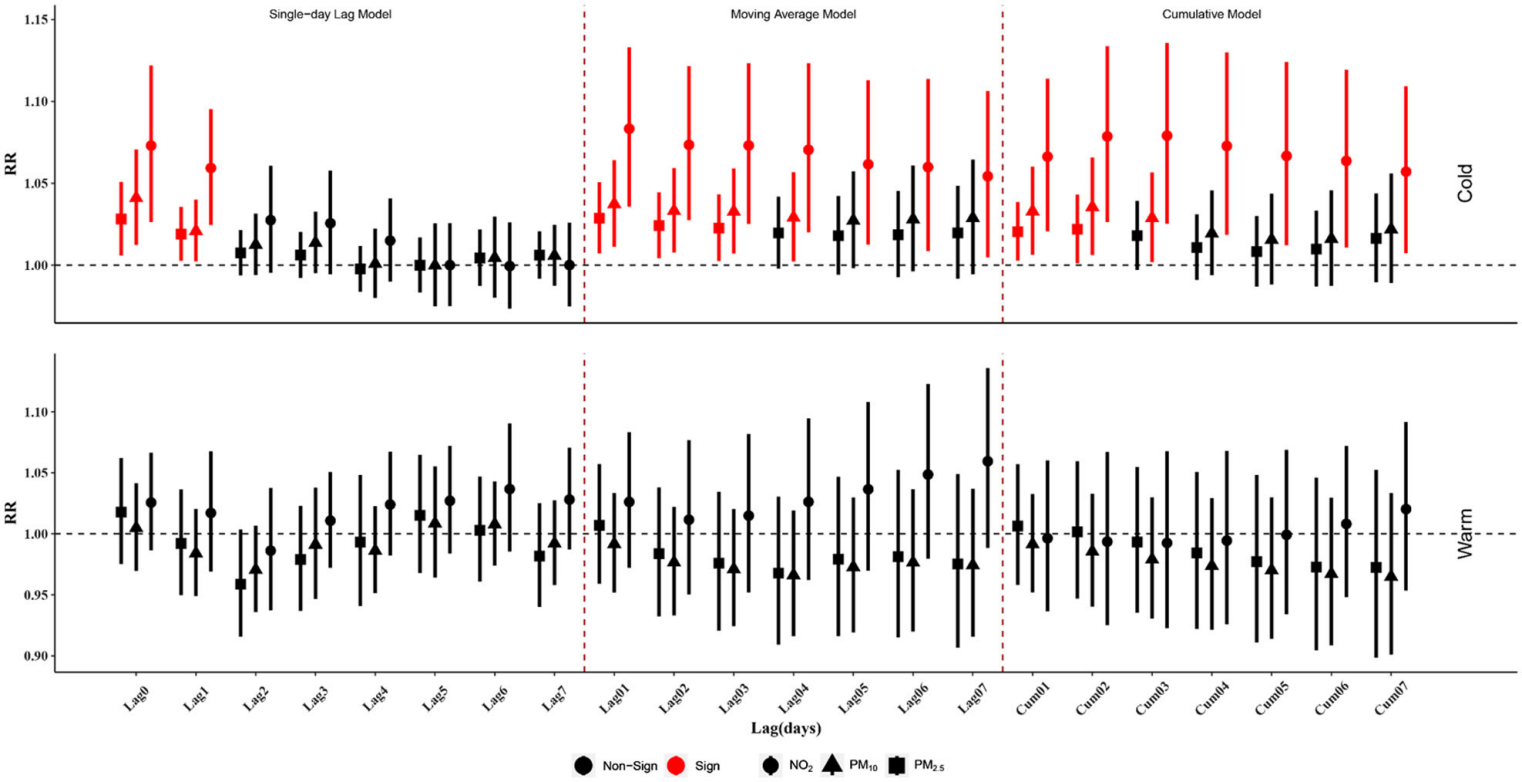
Estimated relative risks (RRs) and 95% confidence intervals (CIs) of PTB for an IQR increase of PM_2.5_, PM_10_ and NO_2_ in the cold (November to March) and warm (April to October) seasons, in Xi’an, China from 2015 to 2018.

Figure 3 shows the subgroup analysis results of cold and warm season. For the cold season, in the single-day lag model, maternal exposure to PM_2.5_ (Lag0: RR: 1.028, 95%CI: 1.006–1.051; Lag1: RR: 1.019, 95%CI: 1.003–1.036), PM_10_ (Lag0: RR: 1.041, 95%CI: 1.012–1.071; Lag1: RR: 1.021, 95%CI: 1.002–1.040) and NO_2_ (Lag0: RR: 1.073, 95%CI: 1.026–1.122; Lag1: RR: 1.059, 95%CI: 1.025–1.095) was significant associated with an increased risk of PTB at Lag0 and Lag1. In the moving average model, maternal exposure to PM_2.5_ was significant associated with an increased risk of PTB at Lag01 to Lag03 with the maximum RR of 1.029 (1.007, 1.051) at Lag01, and maternal exposure to PM_10_ was significant associated with an increased risk of PTB at Lag01 to Lag04 with the maximum RR of 1.037 (1.011, 1.064) at Lag01, and maternal exposure to NO_2_ was significant associated with an increased risk of PTB at Lag01 to Lag07 with the maximum RR of 1.083 (1.036, 1.133) at Lag01. In the cumulative model, PM_2.5_ and PM_10_ were significantly associated with an increased risk of PTB at Lag01 to Lag02 and Lag01 to Lag03 with the maximum RR of 1.022 (1.001, 1.043) and 1.036 (1.006, 1.066) at Lag02, respectively. Maternal exposure to NO_2_ was significant associated with an increased risk of PTB at Cum01 to Cum07 with the maximum RR of 1.079 (1.025, 1.136) at Cum03. For the warm season, there was no significant association between PM_2.5_, PM_10_ and NO_2_ with PTB.

Table 2 shows the RRs (95% CIs) of PTB at Lag0 to Lag3 and Lag01 to Lag03 in the double-pollutant model. As described in the methods section, we avoided both PM_2.5_ and PM_10_ in the same model due to their correlation coefficient was 0.87. Overall, the results of the double-pollutant models were similar with the single-pollutant model of Lag0 to Lag3 and Lag01 to Lag03. The significance of NO_2_ remained robust after the adjustment of PM_2.5_ and PM_10_, respectively, but the effect estimates of NO_2_ slight changed. When NO_2_ was adjusted by PM_2.5_ in the double-pollutant model at Lag01, the effects became insignificant.

**Table 2.** Estimated relative risks (RRs) and 95% confidence intervals (CIs) of double-pollutant models.

Pollutants	Lag0	Lag1	Lag2	Lag3	Lag01	Lag02	Lag03
NO_2_	1.021 (0.999, 1.0425)	1.025 (1.003, 1.047)*	1.005 (0.982, 1.028)	1.009 (0.989, 1.0295)	1.029 (1.004, 1.054)*	1.027 (0.998, 1.056)	1.024 (0.997, 1.053)
+PM_2.5_	1.017 (0.992, 1.042)	1.027 (1.001, 1.054)*	1.009 (0.983, 1.036)	1.017 (0.9936, 1.0407)	1.029 (1.000, 1.060)	1.029 (0.994, 1.065)	1.033 (0.997, 1.070)
+PM_10_	1.016 (0.992, 1.041)	1.032 (1.005, 1.059)*	1.011 (0.981, 1.041)	1.014 (0.991, 1.037)	1.028 (1.001, 1.057)*	1.031 (0.995, 1.068)	1.031 (0.996, 1.068)

*Note*. *p < .05.

The results of sensitivity analyses were similar with the primary analysis (Supplemental Figures S3–S7). When excluding GA less than 24 weeks and 28 weeks, respectively, maternal exposure to NO_2_ was significant associated with an increased risk of PTB, with an IQR increase at Lag0 and Lag1. When excluding maternal age less than 20 years and older than 40 years, maternal exposure to NO_2_ was significant associated with an increased risk of PTB, with an IQR increase at Lag1. When assessing the air pollution exposure by the mean concentration of Xi’an and IDW with powers of 1, respectively, the association between NO_2_ and PTB remained significant.

## Discussion

In this study, we examined the association between maternal exposure to PM_2.5_, PM_10_ and NO_2_ during the last seven days of pregnancy and PTB based on DLM and GAM, using more than 400,000 births from 2015 to 2018 in Xi’an city. Our results showed that in the single-day lag model, moving average model, and cumulative model, maternal exposure to NO_2_ might increase the risk of PTB at Lag1, Lag01 and Cum01 to Cum03 (Figure 2). The effects between PM_2.5_, PM_10_ and NO_2_ and PTB are more significant in the cold season (Figure 3). This conclusion of single-day lag model and moving average model still held true after adjusting for PM_2.5_ and PM_10_. The results of a variety of sensitivity analyses were similar to the primary analysis.

Our results found that the effect of NO_2_ on PTB was relatively robust and PM_2.5_ and PM_10_ showed a significant effect under modification of seasons. Several previous studies have reported the relationship between air pollution and PTB in the week before delivery. For instance, Liu et al. found that the lag effect of NO_2_, PM_2.5_ and PM_10_ reached a peak at day 3 before delivery with per IQR increase [28]. Our results showed that the lag effect reached a peak at days 0 and 1 before delivery. Similarly, Siddika et al. found that the risk of PTB was related to exposures to PM_2.5_, PM_10_ and NO_2_ during the week before delivery and these associations were present mainly in the cold season in Finland between 1984 and 1990 [29]. This was broadly consistent with our results of Lag07 in the cold season, maternal exposure to NO_2_ was significant associated with an increased risk of PTB. Zhao et al. observed the risk of PTB increased with an IQR increase in NO_2_ at Lag0 [20], which was consistent with our findings. Zhao et al. and Guan et al. showed maternal exposure to PM_10_ and PM_2.5_ was a risk factor at Lag0 and Lag3, respectively [20,30], which was similar with our results in the cold seasons. In addition, Guan et al. found the risk of PM_2.5_ for acute effects during warm seasons was higher than during cold seasons [30], which was contrary to our results, our subgroup analysis suggested that the cold season has a greater effect on PTB than the warm season. The considerable differences in the particulate matter compositions and sources between cold and warm seasons in Xi’an may account for this finding. Further research is required to explore underlying reasons. The study conducted by Sagiv et al. in Pennsylvania during 1997–2001 found an acute effect of exposure to PM_10_ in two days and five days before delivery on the risk of PTB with no statistically significant difference [18], in addition, some studies have found PTB were positive related with air pollution, while the effects were no statistically significant [19,21,31,32]. This may be related to the low level of air pollution in the study area. Li et al. found that in the adjusted single-pollutant city-specific model, the association between short-term air pollutants (PM_2.5_, PM_10_ and NO_2_) exposure and PTB was significant, but in the two cities model, there was no statistically significant [33]. This may be related to the misclassification of exposure. Due to the distribution of air pollution in a city is uneven, and the average value of air pollution in a city may not be accurate enough. Differences between our study and previous studies may be attributed to differences in the level of air pollution, population differences, statistical methods, and so on, especially for the selection of the analysis model was quite different.

The mechanism by which air pollutant triggered PTB is unclear but may be related to inflammation or oxidative stress [34–37]. A review indicated that the effects of air pollution on PTB may manifest through the cardiovascular mechanisms of oxidative stress, inflammation, coagulation, endothelial function, and hemodynamic responses [38]. An animal study showed that the third trimester is more susceptible to air pollution exposures for PTB, consistent with increased effects estimated in the last seven days of pregnancy in our study [39]. In addition, that black carbon particles can reach the foetal side of the human placenta, which may be a potential mechanism of the observed adverse effects of air pollution [40]. Hormones may also play an intermediate role between air pollution and PTB [40–42].

There are some limitations in our study. First, the measurements from stationary outdoor monitors may not enough represent individual exposure, and it may introduce exposure misestimate by not accounting for maternal mobility in pregnancy and estimating air pollution exposure at the county level. And our air pollution assessment accuracy was districts and counties, rather than cities commonly used in previous studies. While there may be a large variation in air pollution over a city, air pollution is relatively homogeneous within a given county, and ambient air pollution is a strong proxy of personal exposure [43]. As far as we know, we are one of the few studies that have used two-stage analysis at the city level to more accurately assess the short-term effects of extreme air pollution exposure on PTB. Second, given that demographic information about PTB was unavailable in our study, our results cannot adjust for these confounders. But our population-based study reduces the selection bias, and the long study period and large population enhanced the precision of the estimated effect. Third, we did not exclude enough medically preterm birth, while given the relatively uniform distribution of PTB unrelated to air pollution over time, the real temporal association between air pollutant and PTB would not be greatly affected even if this part of PTB were not excluded. Finally, the daily count of PTB in some counties is few, which may impact the statistical power to detect significant associations in the first stage.

PTB is a crucial global health problem, and addressing it is critical to reduce global infant and child mortality and achieving the Sustainable Development Goals. In a population study, Xu et al. observed greater combined effects of multiple pollutants in female than in male adults [44]. Therefore, during this particular period of pregnancy, pregnant women are exposed to air pollution is more dangerous. Previous studies combined with our findings highlight that pregnant women are a susceptible population who should be considered central to the public health response to air pollution. Additional high-quality research is required to further clarify the short-term effects of air pollution and preterm birth.

## Conclusions

Based on an analysis of more than four hundred thousand births over four years in Xi’an city, we observed maternal exposure to NO_2_, PM_2.5_ and PM_10_ before delivery have a significant association risk for PTB, particularly in the cold season. These findings can provide more epidemiological evidence for exploring the relationship between air pollution and PTB and can provide a basis for future policy formulation regarding air pollution and PTB.

## Supplementary Material

Supplemental Material

## Data Availability

The data that support the findings of this study are available on request from the corresponding author, WFY.
