# Supplementary material for: Short-term effects of air pollution exposure on the risk of preterm birth in Xi’an, China
Source: Ann Med. 2023 Jan 4;55(1):325–34. doi: 10.1080/07853890.2022.2163282 (PMC9828631; doi:10.1080/07853890.2022.2163282)
Supplement: Supplemental Material [file IANN_A_2163282_SM8391.docx]

Appendix A. Supplementary data


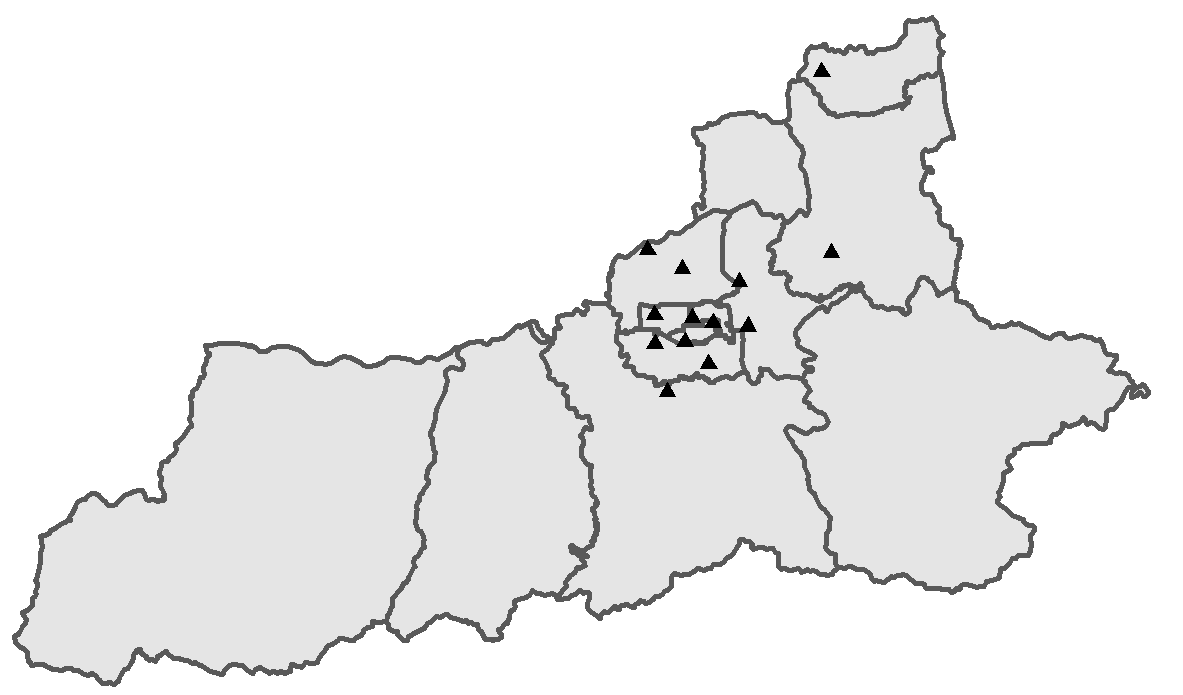


**Supplemental Figure S1.** The map of 13 districts/counties and 13 monitoring stations included in this study (The black triangle represents the monitoring site. Due to geographical characteristics, urban population is mainly concentrated in urban areas.).


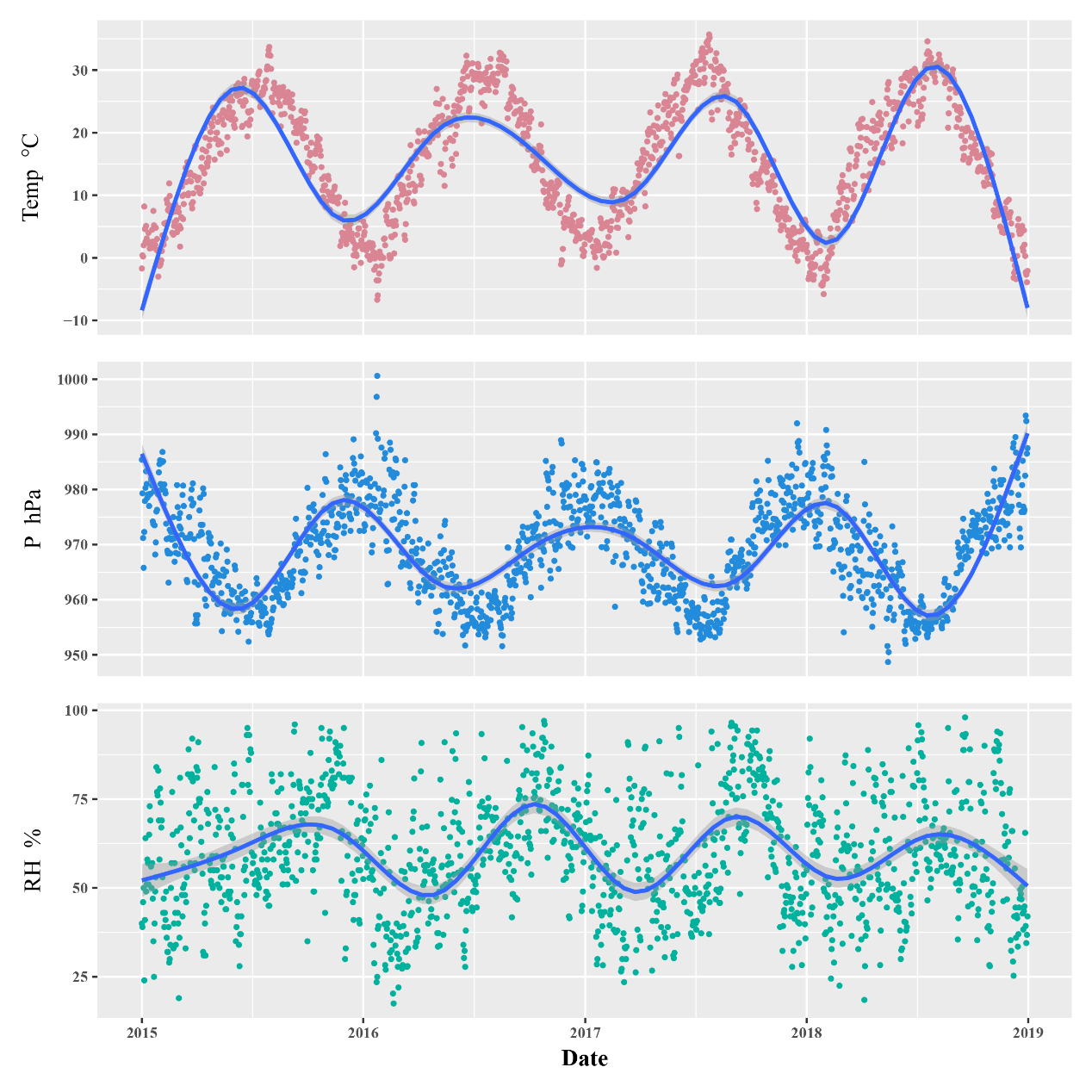


**Supplemental Figure S2.** The distribution of meteorological factors in Xi'an, China during 2015–2018.

**Supplemental Table S1.** The I^2^ of pooled estimate for single-day lag model, moving average model and cumulative model.

| Lag(days) | I^2^ | | | | |
| --- | --- | --- | --- | --- | --- |
|  | PM2.5 |  | PM10 |  | NO2 |
| Lag0 | 34.082 |  | 48.894 |  | 0 |
| Lag1 | 0.355 |  | 0 |  | 6.316 |
| Lag2 | 0 |  | 0 |  | 16.976 |
| Lag3 | 0 |  | 11.924 |  | 0 |
| Lag4 | 0 |  | 17.509 |  | 0 |
| Lag5 | 31.209 |  | 49.567 |  | 0 |
| Lag6 | 15.539 |  | 41.29 |  | 0 |
| Lag7 | 0 |  | 0 |  | 0 |
| Lag01 | 23.265 |  | 28.226 |  | 5.859 |
| Lag02 | 19.401 |  | 23.167 |  | 16.957 |
| Lag03 | 20.161 |  | 27.292 |  | 6.606 |
| Lag04 | 24.335 |  | 30.784 |  | 3.732 |
| Lag05 | 31.347 |  | 32.928 |  | 0 |
| Lag06 | 36.263 |  | 40.022 |  | 0 |
| Lag07 | 37.155 |  | 41.301 |  | 0 |
| Cum01 | 0 |  | 11.577 |  | 0 |
| Cum02 | 0 |  | 13.958 |  | 0.804 |
| Cum03 | 0 |  | 6.496 |  | 10.687 |
| Cum04 | 0 |  | 0 |  | 8.188 |
| Cum05 | 0 |  | 0 |  | 0 |
| Cum06 | 7.179 |  | 0 |  | 0 |
| Cum07 | 15.0.35 |  | 0 |  | 0 |


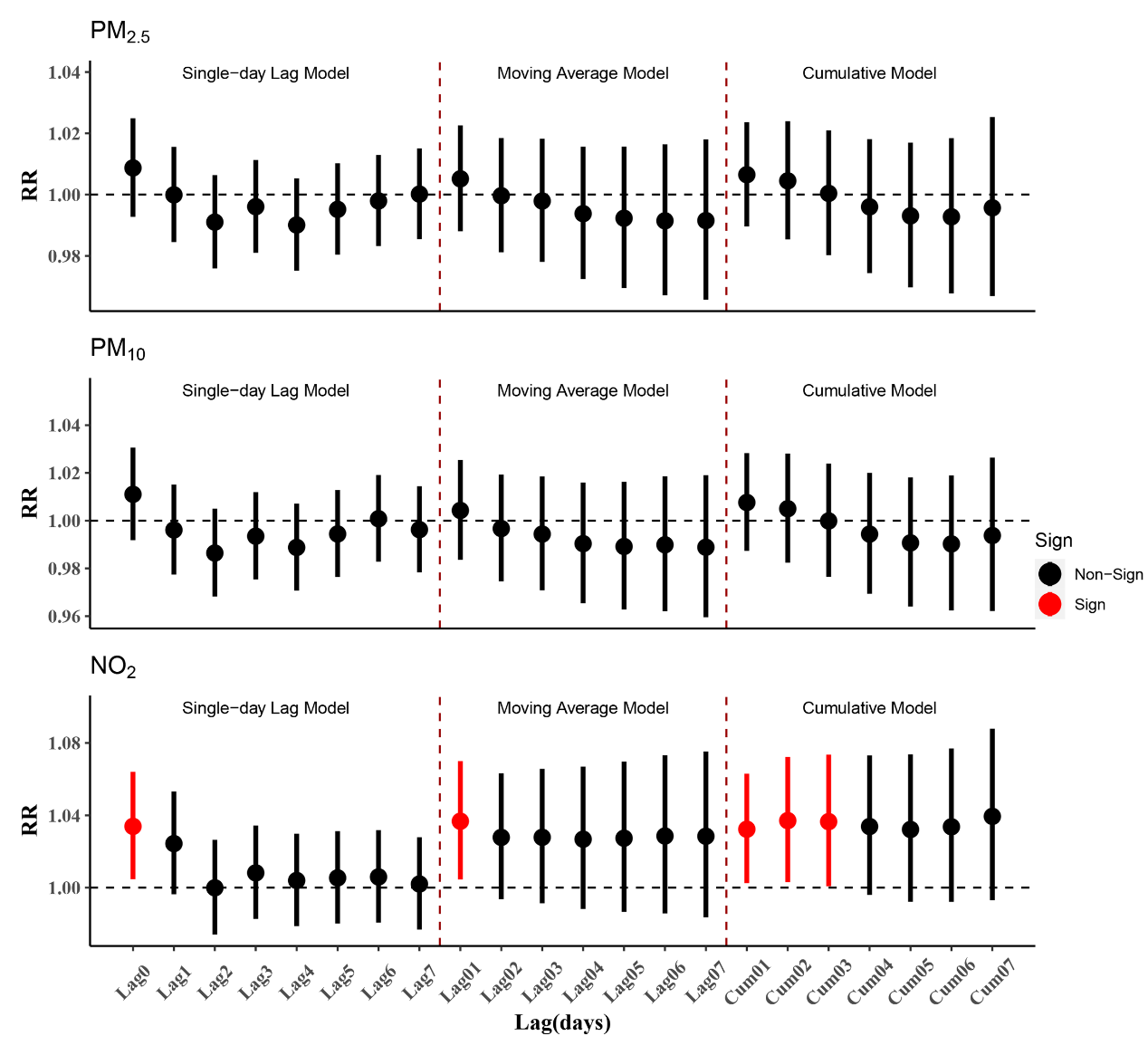


**Supplemental Figure** S**3**. Estimated relative risks (RRs) and 95% confidence intervals (CIs) of PTB for an IQR increase of PM_2.5_, PM_10_ and NO_2_ of mean concentration of Xi'an.
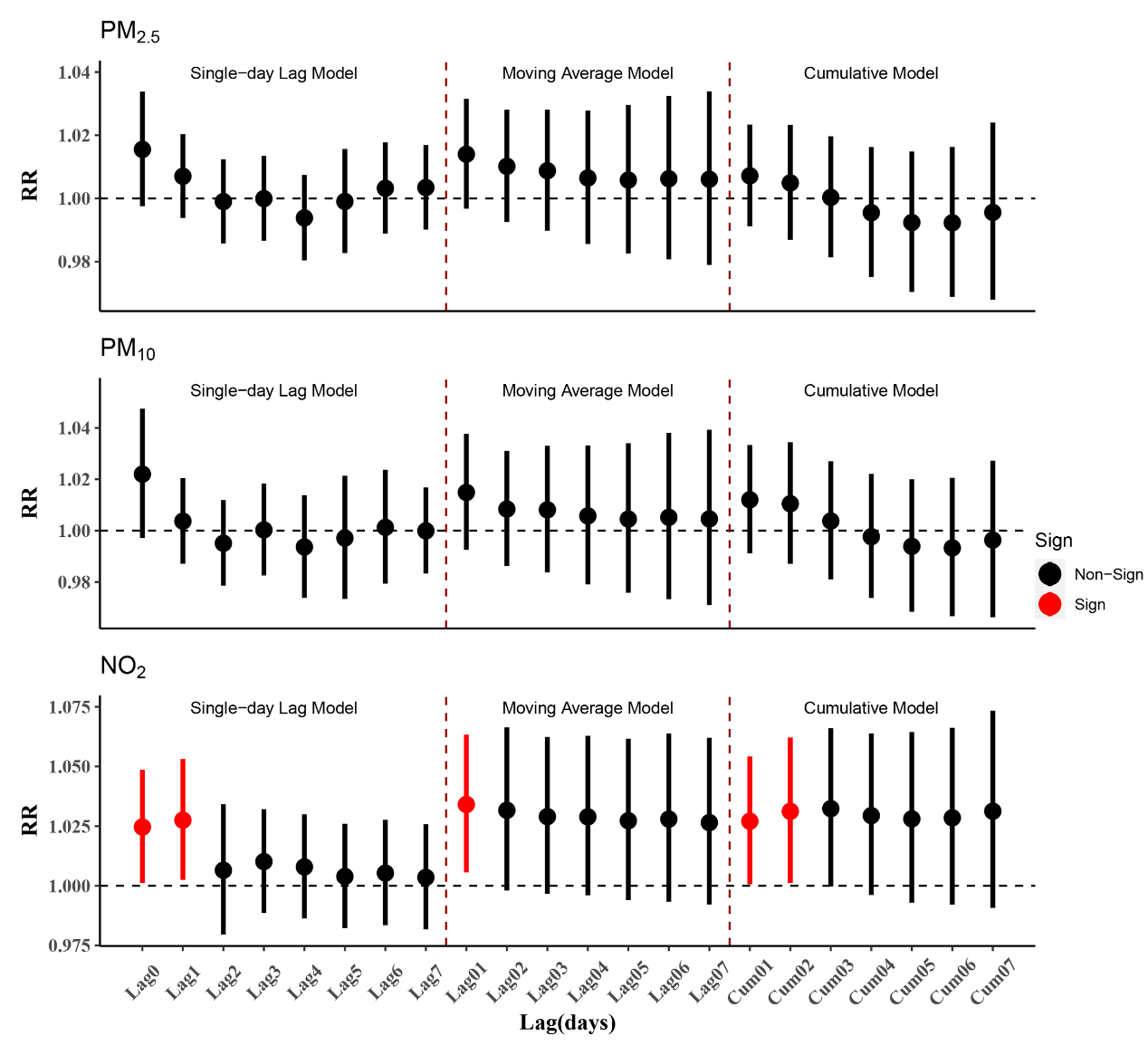


**Supplemental Figure S4.** Estimated relative risks (RRs) and 95% confidence intervals (CIs) of PTB for an IQR increase of PM_2.5_, PM_10_ and NO_2_ assessed by IDW with powers of 1.
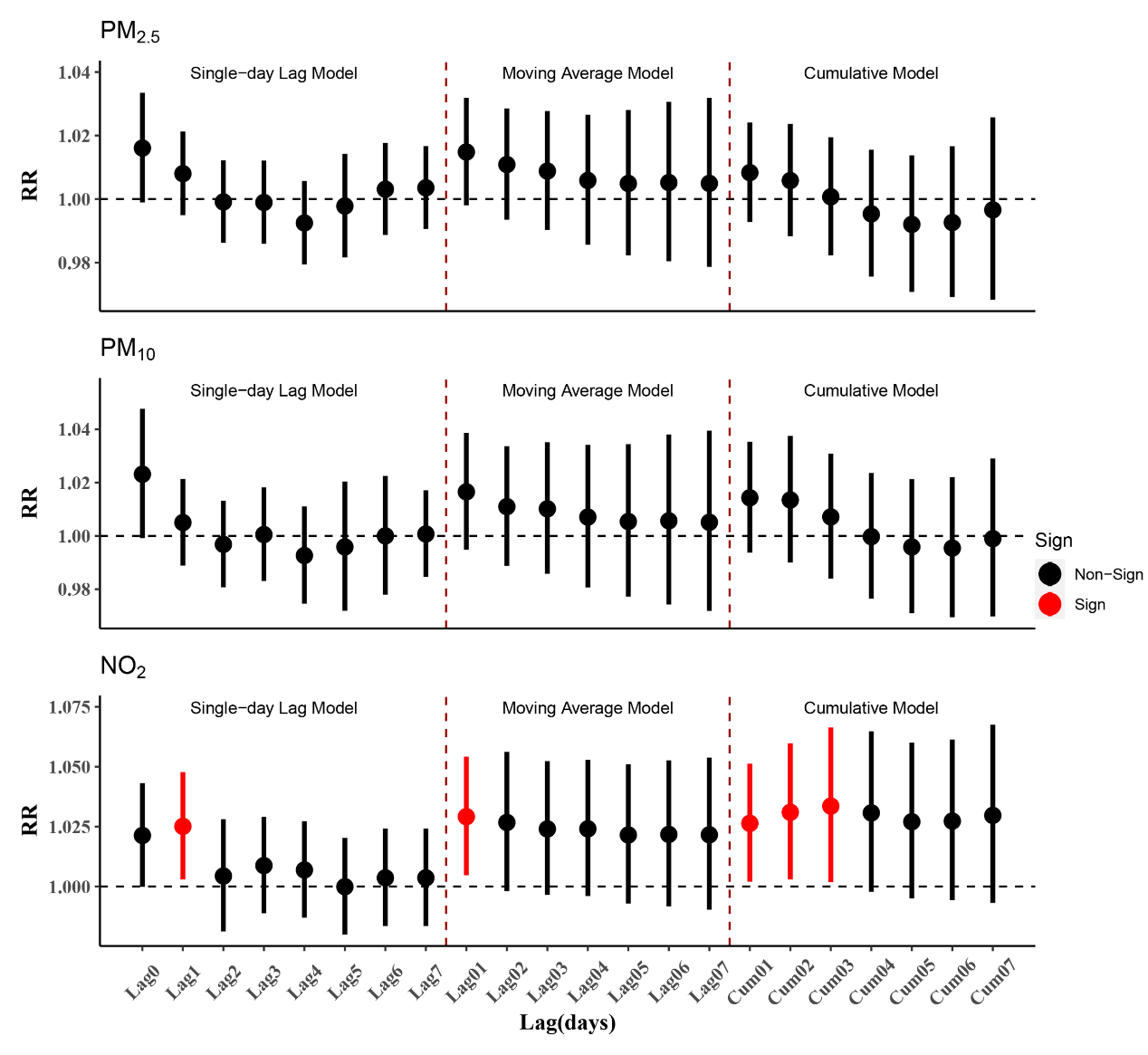


**Supplemental Figure S5.** Estimated relative risks (RRs) and 95% confidence intervals (CIs) of PTB (GA ≥ 24 weeks) for an IQR increase of PM_2.5_, PM_10_ and NO_2_.
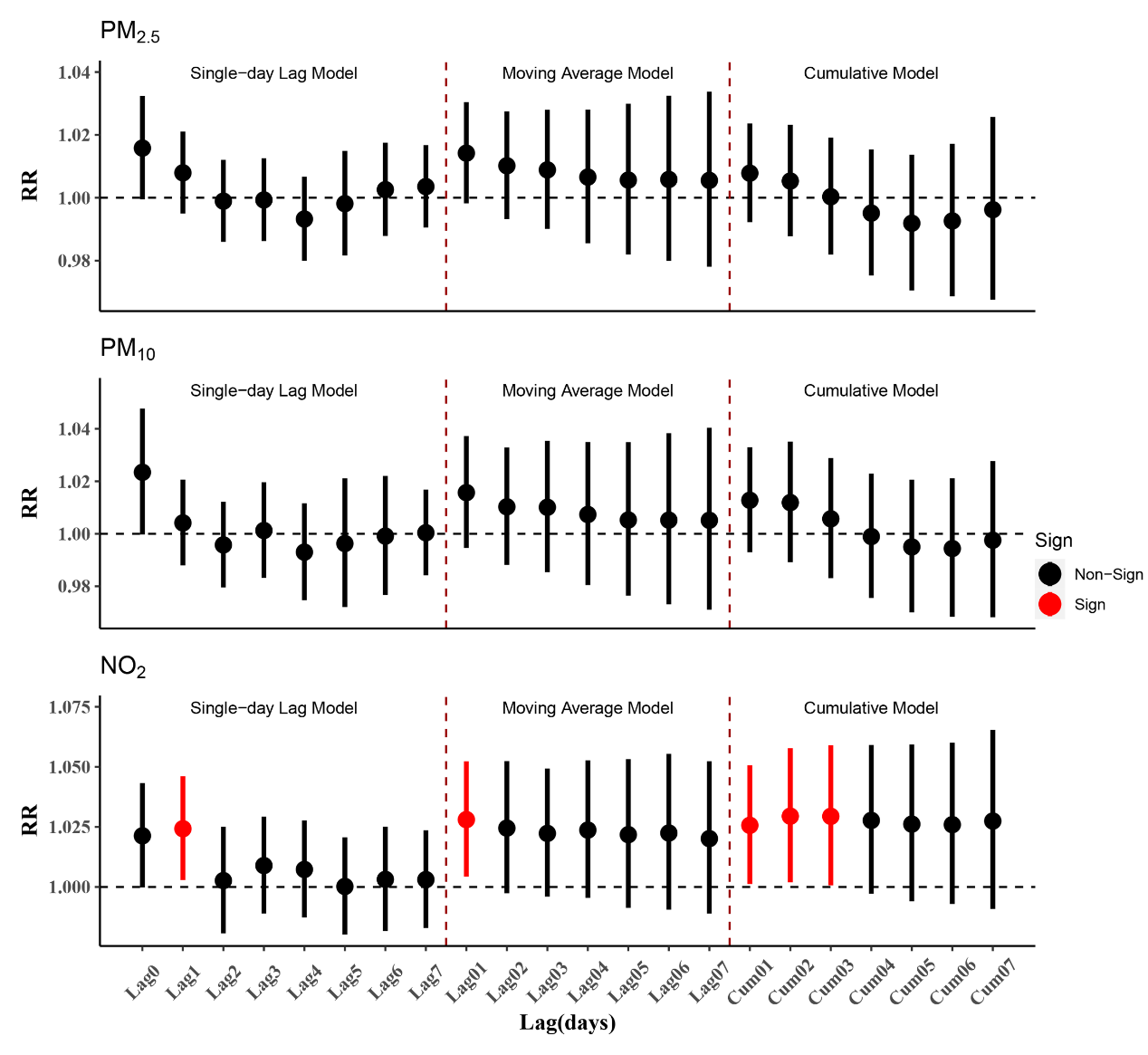


**Supplemental Figure S6**. Estimated relative risks (RRs) and 95% confidence intervals (CIs) of PTB (GA ≥ 28 weeks) for an IQR increase of PM_2.5_, PM_10_ and NO_2_.
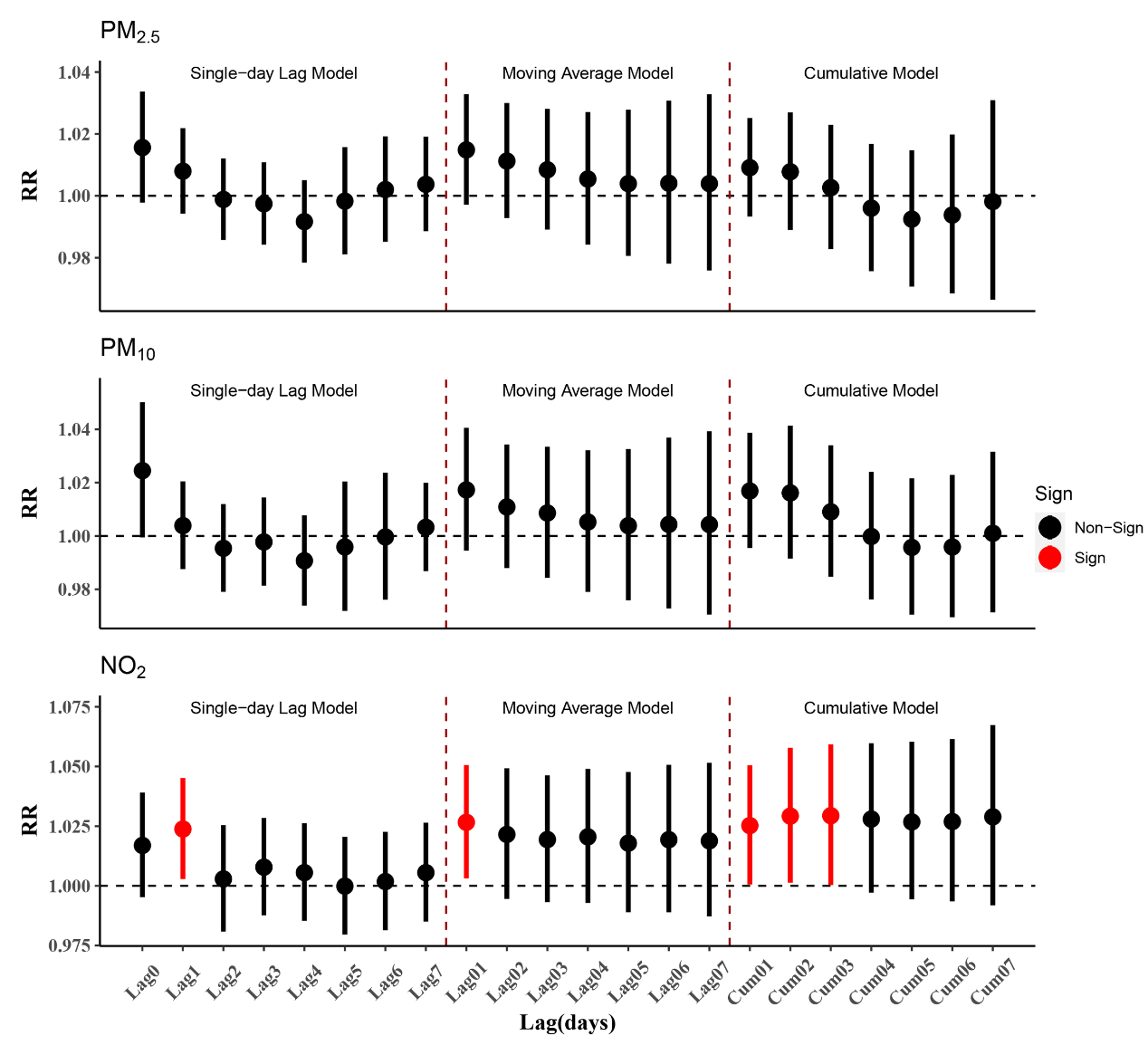


**Supplemental Figure S7.** Estimated relative risks (RRs) and 95% confidence intervals (CIs) of PTB (20 years ≤ maternal age ≤ 40 years) for an IQR increase of PM_2.5_, PM_10_ and NO_2_.
